# Impact of renal denervation on circadian variations of blood pressure and clock gene expression in spontaneously hypertensive rats

**DOI:** 10.3389/fcvm.2026.1739153

**Published:** 2026-04-01

**Authors:** Guan-Ying Yang, Shi-Kun Chen, Si-Qi Luo, Jin-Zi Su, Wen-Qin Cai

**Affiliations:** 1 Department of Geriatrics, The First Affiliated Hospital of Fujian Medical University, Fuzhou, China; 2 Department of Rehabilitation with Traditional Chinese Medicine of Jiangbei Campus, The First Affiliated Hospital of Army Medical University, Chongqing, China; 3 Department of Rehabilitation Medicine, The School of Health, Fujian Medical University, Fuzhou, China; 4 Fujian Hypertension Research Institute, The First Affiliated Hospital of Fujian Medical University, Fuzhou, China; 5 Department of Rehabilitation Medicine, The First Affiliated Hospital of Fujian Medical University, Fuzhou, China; 6 Department of Cardiology, The First Affiliated Hospital of Fujian Medical University, Fuzhou, China; 7 Department of Cardiology, National Regional Medical Center, Binhai Campus of the First Affiliated Hospital, Fujian Medical University, Fuzhou, China; 8 Department of Geriatrics, National Regional Medical Center, Binhai Campus of the First Affiliated Hospital, Fujian Medical University, Fuzhou, China; 9 Clinical Research Center for Geriatric Hypertension Disease of Fujian Province, The First Affiliated Hospital of Fujian Medical University, Fuzhou, China

**Keywords:** BMAL1, circadian variation, hypertension, RAS system, renal denervation

## Abstract

**Background:**

Hypertension is often associated with elevated nighttime blood pressure and is a significant risk factor for cardiovascular and cerebrovascular diseases. This study explored the effects of renal denervation (RDN) on circadian blood pressure rhythms and clock gene expression in spontaneously hypertensive rats (SHRs).

**Methods:**

Ten-week-old SHRs were randomized into RDN and sham surgery (Sham) groups, with Wistar-Kyoto (WKY) rats as controls. Blood pressure was measured at rest (14:00) and during activity (02:00) biweekly, and blood pressure variability was analyzed.

**Results:**

RDN significantly reduced BP and enhanced circadian BP variation, particularly during the resting phase. The Sham group displayed minimal circadian variations in plasma and renal norepinephrine levels, whereas the RDN group exhibited an overall reduction in norepinephrine levels, with lower levels at rest than during activity. Furthermore, the Sham group showed no significant circadian variation in the renin-angiotensin-aldosterone system (RAS), whereas RDN restored circadian rhythms in ACE1, Ang II, ACE2, and Ang1-7. Additionally, the Sham group demonstrated consistently high renal BMAL1 protein expression throughout the day, whereas RDN reduced BMAL1 expression during the resting phase, indicating restored circadian variation.

**Conclusions:**

These findings suggest that RDN not only lowers blood pressure but also improves circadian rhythm, likely through the modulation of sympathetic nervous activity, the RAS system, and the circadian clock gene BMAL1.

## Background

Hypertension is a predominant risk factor contributing to the global incidence and mortality associated with cardiovascular diseases (1). Epidemiological data indicate that approximately 33% of the world's population, equivalent to 2.64 billion individuals out of 8 billion, is affected by hypertension, thereby establishing it as a substantial global public health concern (2). The physiological blood pressure rhythm typically manifests as an elevation upon morning awakening, maintenance during diurnal activity, and subsequent nocturnal reduction (with nighttime blood pressure averaging 10%–20% lower than daytime measurements) (3). Based on nocturnal blood pressure variations, circadian blood pressure patterns can be classified into four distinct categories: dipper (nocturnal blood pressure reduction > 10%), extreme dipper (nocturnal reduction > 20%), non-dipper (nocturnal reduction ≤ 10%), and reverse dipper (nocturnal blood pressure exceeding daytime levels) (4). Clinical observations have demonstrated a higher prevalence of abnormal blood pressure rhythms among hypertensive patients (5), particularly in cases of resistant hypertension, where non-dipper patterns are more pronounced (6). Notably, non-dipper hypertensive patients exhibit more severe target organ damage than their dipper counterparts (7, 8).

Aryl hydrocarbon receptor nuclear translocator-like protein (BMAL1), alternatively designated Arntl3 in murine models and MOP3 in humans, serves as a principal regulator of the mammalian molecular clock (9). Genetic ablation of BMAL1 has been shown to disrupt 24-hour activity patterns, a fundamental measure of circadian output (10). Peripheral circadian clock genes demonstrate tissue-specific expression patterns, with aortic BMAL1 playing a crucial role in circadian regulation of blood pressure and heart rate (11). Furthermore, BMAL1 in perivascular adipose tissue modulates resting-phase blood pressure through transcriptional regulation of angiotensinogen (12). However, the specific role of renal BMAL1 in circadian blood pressure regulation remains to be fully elucidated, despite the importance of the kidney in blood pressure homeostasis.

Beyond genetic regulation, the sympathetic nervous system contributes significantly to circadian modulation of blood pressure (13). Scientific evidence has confirmed the association between elevated morning blood pressure and sympathetic nervous system activity (14). In patients with primary hypertension, nocturnal norepinephrine levels exhibit characteristic elevations during sleep (15). Renal denervation (RDN) has recently emerged as a promising therapeutic intervention for hypertension, primarily through the modulation of sympathetic nervous system activity (16). In rat models of metabolic syndrome, RDN has been shown to restored normal circadian blood pressure patterns (17). Nevertheless, the efficacy of RDN in regulating circadian blood pressure in spontaneously hypertensive rats remains to be determined. The present study aimed to investigate the effects of RDN on circadian blood pressure regulation and expression patterns of the core clock gene BMAL1 in spontaneously hypertensive rats.

## Methods

### Animals

Eight-week-old male spontaneously hypertensive rats (SHRs) and age-matched Wistar-Kyoto (WKY) rats were purchased from the Vital River Laboratory Animal Technology Company (Beijing, China), with the animal experiment quality certificate number SCXK (Jing) 2021-0006. The animals were housed in a specific pathogen-free (SPF) barrier environment at the Experimental Animal Center of Fujian Medical University under controlled temperature and humidity conditions, with standard feed and free access to food and water. The laboratory used artificial lighting with a 12-h light/12-h dark cycle (lights on from 08:00 to 20:00). All animal handling procedures complied with the regulations set forth by the Animal Ethics Committee of Fujian Medical University and were approved by the Experimental Animal Ethics Committee of Fujian Medical University, (ethical review number IACUCFJMU2022-0569).

### Surgical procedures

Following a 2-week period of adaptive feeding, SHRs were randomly assigned to either the renal denervation (RDN) group or the sham surgery group. The rats were anesthetized via intraperitoneal injection of carbamate (800 mg/kg) and α-chloralose (40 mg/kg). A dorsal-lateral incision was made to expose the renal artery and the vein sheath. Under a dissecting microscope, the renal artery and vein were carefully isolated from the surrounding connective tissue, and the visible nerve fibers were severed. Sterile gauze soaked in a 10% phenol- ethanol solution was applied to the renal vessels for 10 min to perform renal sympathetic nerve ablation, while the sham group received saline application to the renal artery, and WKY rats underwent no treatment. The contralateral side was treated in a similar manner.

### Measurement of blood pressure

Non-invasive tail-cuff plethysmography (BP-300, Chengdu Taimeng Science and Technology Company, China) was used to measure the resting (14:00) and active (02:00) systolic blood pressure (SBP) of each rat both preoperatively and biweekly postoperatively until the conclusion of the experiment (16 weeks of age). The study indicated that rats exhibited lower blood pressure during their resting phase at 14:00, whereas elevated blood pressure was observed during their active phase at 02:00 (18). Blood pressure variability was calculated using the following formula: variability = (active period blood pressure–resting period blood pressure)/active period blood pressure. To record pulsations of the tail artery, the rats were kept in a thermostatic chamber at 30 °C for 10 min before each measurement, averaged from three readings.

### Sample collection and preservation

After measuring blood pressure at 16 weeks of age, tissue samples were collected from each group of rats at 14:00 and 02:00. The specific procedure was as follows: the rats were deeply anesthetized by intraperitoneal injection of a mixture of carbamate (800 mg/kg) and α-chloralose (40 mg/kg). Following blood collection from the abdominal aorta, the rats were euthanized by cervical dislocation under deep anesthesia and the kidneys were harvested. One kidney was fixed in formaldehyde, while the other was rapidly frozen in liquid nitrogen and subsequently stored at −80 °C for future analysis. Plasma samples were isolated from whole blood and stored at −80 °C.

### ELISA for norepinephrine (NE), angiotensin II (Ang II) and angiotensin1-7 (Ang1-7) levels

Samples (50) mg were taken from the renal cortex and added to 0.5 mL of phosphate-buffered saline (PBS solution). The mixture was homogenized using an electric homogenizer at 7,000 rpm for 15 s; and this procedure was repeated three times. The obtained homogenate was centrifuged at 3,000 rpm for 20 min at 4 °C, and the supernatant was collected. NE ELISA kits (YJ1002827), Ang II ELISA kits (YJ002823), and Ang1-7 ELISA kits (YJ920741) from the Enzyme-Link Biotechnology Company in China were used to measure the NE, Ang II, and Ang1-7 content, following the manufacturer's instructions outlined in the kit manual.

### Western blot analysis

Kidney cortex tissues were ground using RIPA lysis buffer (P0013B, Beyotime, China) containing protease inhibitors (P1050, beyotime, China), and protein concentrations were determined using the BCA Protein Concentration Measurement Kit (AR1189, BOSTER, China) to determine protein concentration. Protein expression was analyzed by western blotting, as described previously (19). Antibodies and dilutions were as follows: ACE1, 1:1000 dilution (A11357, ABclonal, China); ACE2, 1:1000 dilution (A12737, ABclonal, China); AT1R, 1:1000 dilution (25343-1-AP, Proteintech, China) and MasR, 1:800 dilution (sc-390453, Santa Cruz, USA).

### Immunohistochemical (IHC) staining

The kidneys were fixed with paraformaldehyde, embedded in paraffin, and sectioned at a thickness of 4 μm using a slicer. The slides were deparaffinized and incubated with citrate buffer. After treatment with H_2_O_2_ to inhibit endogenous peroxidase, the sections were blocked with diluted BSA, the Tyrosine hydroxylase antibodies were used for IHC staining.

### Statistical analysis

Data analysis was conducted using SPSS 25. Student's *t*-test or one-way ANOVA followed by Fisher's least significant difference (LSD) *post hoc* test was used to assess the differences between groups. All data are presented as mean ± SEM. *P* < 0.05. Active phase (night) of WKY, Sham and RDN groups compared to their respective resting phase (day), a *P* < 0.05; the overall level of Sham group compared to WKY group, b *P* < 0.05; the overall level of RDN group compared to Sham group, c *P* < 0.05.

## Results

### Impact of RDN on circadian variation of blood pressure

At the baseline assessment conducted at 10 weeks of age, no statistically significant differences in systolic blood pressure (SBP) were observed between the sham and RDN groups during either the resting or active phases. However, both experimental groups demonstrated significantly elevated SBP values compared with the WKY control group. RDN intervention resulted in a significant reduction of SBP in SHRs during both circadian phases, with maximal therapeutic efficacy observed at 12 weeks post-intervention. Comprehensive quantitative data are presented in Tables 1, 2.

**Table 1:** Changes in resting systolic blood pressure (SBP/mm Hg) (*n* = 8).

Age (weeks)	WKY	Sham	RDN
10	129.9 ± 7.6	176.8 ± 6.9^b^	178.1 ± 6.7^b^
12	128.5 ± 4.8	181.9 ± 9.5^b^	145.0 ± 7.1^b,c^
14	132.6 ± 4.4	196.4 ± 9.7^b^	151.2 ± 6.9^b,c^
16	131.0 ± 5.2	197.8 ± 7.6^b^	152.0 ± 6.8^b,c^

1 mm Hg = 0.133 kPa.

All data are presented as mean ± SEM. *P*<0.05 was considered statistically significant. the overall level of Sham group or RDN group compared to WKY group, ^b^*P* < 0.05; the overall level of RDN group compared to Sham group, ^c^*P* < 0.05.

**Table 2:** Changes in active systolic blood pressure (SBP/mm Hg) (*n* = 8).

Age (weeks)	WKY	Sham	RDN
10	145.1 ± 6.8	189.4 ± 8.3^b^	190.0 ± 9.3^c^
12	141.6 ± 5.6	193.3 ± 11.0^b^	160.2 ± 6.1^b,c^
14	146.8 ± 4.2	207.8 ± 11.9^b^	164.7 ± 5.9^b,c^
16	146.0 ± 5.5	206.5 ± 7.6^b^	166.0 ± 3.6^b,c^

All data are presented as mean ± SEM. *P*<0.05 was considered statistically significant. the overall level of Sham group or RDN group compared to WKY group, ^b^*P* < 0.05; the overall level of RDN group compared to Sham group, ^c^*P* < 0.05.

The WKY control group maintained a stable blood pressure variability of approximately 10% throughout the experimental period. Initial measurements at 10 weeks of age revealed no significant differences in blood pressure variability between the Sham and RDN groups. However, by 12 weeks of age, the RDN group demonstrated a significant enhancement in circadian blood pressure rhythm, exhibiting a variability of 9.05 ± 2.44%, which was markedly higher than that of the Sham group (5.84 ± 1.68%; *P* < 0.05). This improvement in circadian regulation persisted at 16 weeks of age, with the RDN group maintaining a blood pressure variability of 8.49 ± 2.66%, significantly exceeding the Sham group's variability of 4.18 ± 2.83% (*P* < 0.05). The detailed statistical data are presented in Table 3.

**Table 3:** Changes in blood pressure variability (%) (*n* = 8).

Age (weeks)	WKY	Sham	RDN
10	10.50 ± 2.96	6.43 ± 3.09^b^	5.91 ± 2.12^b^
12	9.26 ± 1.19	5.84 ± 1.68^b^	9.05 ± 2.44^c^
14	9.62 ± 2.72	5.42 ± 2.68^b^	8.25 ± 1.40
16	10.26 ± 2.05	4.18 ± 2.83^b^	8.49 ± 2.66^c^

All data are presented as mean ± SEM. *P*<0.05 was considered statistically significant. the overall level of Sham group or RDN group compared to WKY group, ^b^*P* < 0.05; the overall level of RDN group compared to Sham group, ^c^*P* < 0.05.

### Impact of RDN on the circadian variations in sympathetic nerve activity

**Figure 1:**
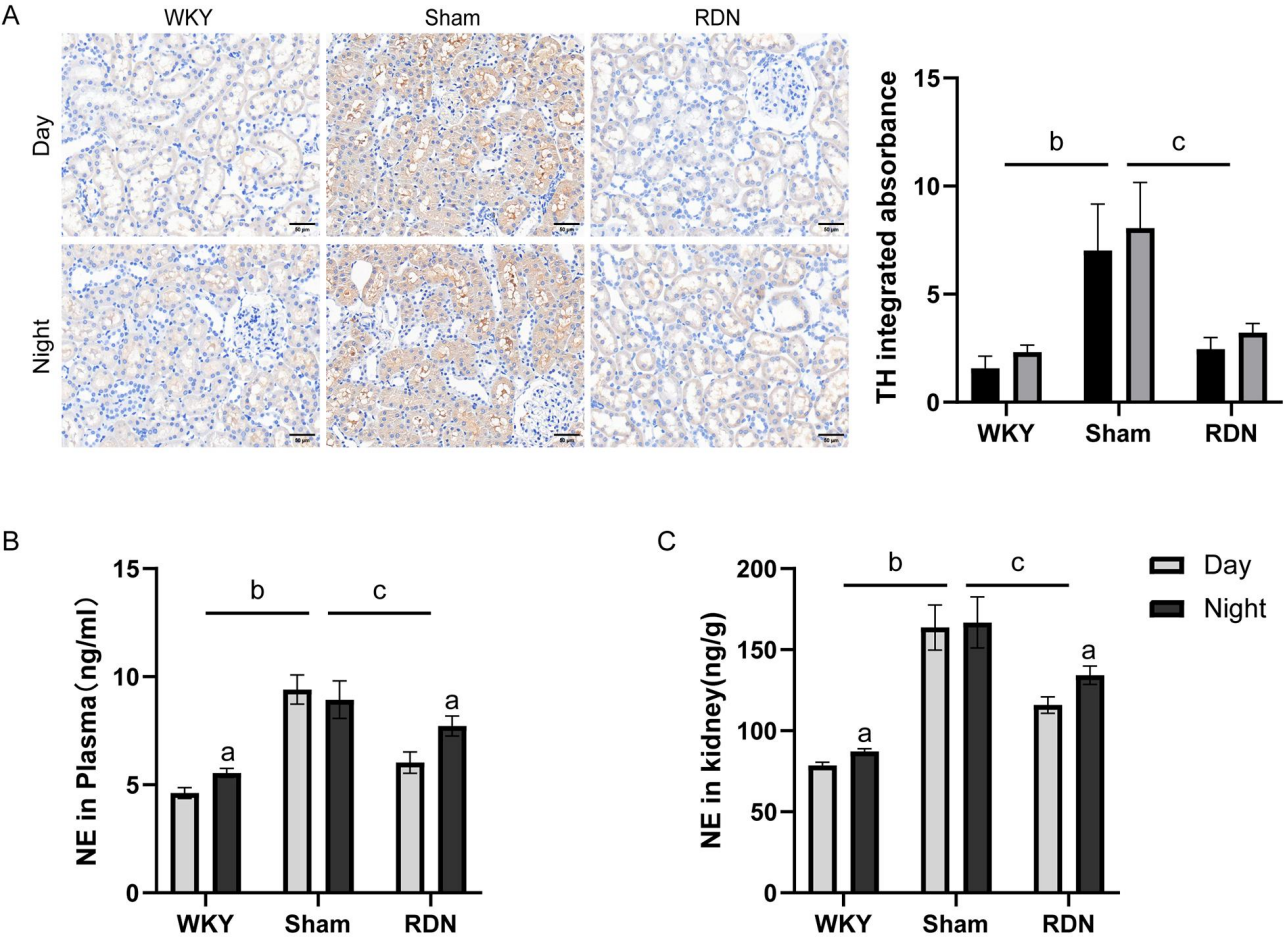
The impact of renal denervation (RDN) on circadian differences in sympathetic nerve activity. **(A)** Immunohistochemical staining of TH, **(B)** the concentration of NE in plasma, **(C)** the concentration of NE in kidney. (**A)** (n=3); **(B,C)** (n=6) Day: resting phase; Night: active phase. Compared to the resting phase (Day), ^a^*P* < 0.05; in comparison to the overall WKY group, ^b^*P* < 0.05; and compare to the overall Sham group, ^c^*P* < 0.05.

Tyrosine hydroxylase (TH), a marker of sympathetic nerve fibers, is a reliable indicator of sympathetic nervous system excitability. Immunohistochemical staining was employed to evaluate TH protein expression in the kidneys of experimental rats during both resting and active phases. The Sham group exhibited elevated TH expression levels during both phases, whereas the RDN group demonstrated an overall reduction in TH expression, with daytime levels significantly lower than night-time levels (Figure 1A).

To further investigate circadian differences in sympathetic nerve activity, norepinephrine (NE) levels were assessed in both renal tissue and plasma. The Sham group exhibited significantly higher overall levels of renal and plasma NE than WKY rats, with no notable circadian variation. In contrast, RDN treatment reduced the overall NE content in hypertensive rats, particularly during the resting phase, thereby enhancing circadian variation in NE levels (Figures 1B,C).

### Impact of RDN on the circadian variations of Ang II and Ang1-7

**Figure 2:**
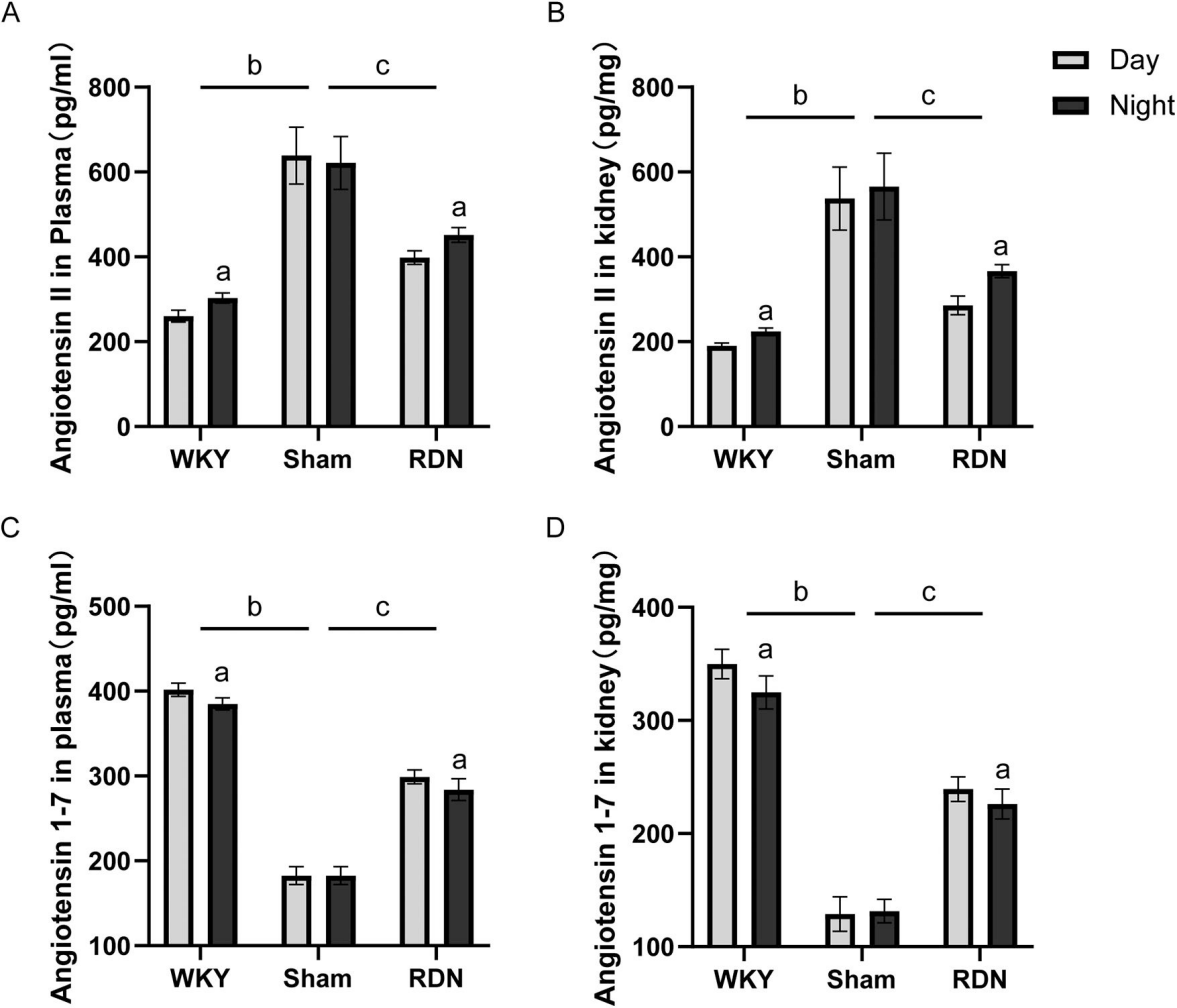
The impact of RDN on the circadian expression differences of renal and plasma Ang II and Ang1-7 (*n*=6). **(A)** Plasma Angiotensin II, **(B)** Renal Angiotensin II, (C) Plasma Angiotensin 1-7, **(D)** Renal Angiotensin 1-7. Day: resting phase, Night: active phase. Compared to the resting phase (Day), ^a^*P* < 0.05; in comparison to the overall WKY group, ^b^*P* < 0.05; and compare to the overall Sham group, ^c^*P* < 0.05.

In WKY rats, both renal and plasma Ang II levels exhibited a distinct circadian rhythm, with significantly elevated levels during the active phase compared with the resting phase. The Sham group displayed higher levels of renal and plasma Ang II than the WKY group, with no discernible circadian variations. RDN effectively reduced Ang II levels, particularly during the resting phase, and restored the circadian rhythm of Ang II expression (Figures 2A,B).

Similarly, WKY rats demonstrated a circadian rhythm in renal and plasma Ang1-7 levels. In the Sham group, no significant circadian variation in Ang1-7 expression was observed, and the overall expression levels were relatively low. Following RDN treatment, SHRs exhibited a pronounced circadian variation in renal and plasma Ang1-7, with levels during the active phase being lower than those during the resting phase, and overall expression surpassing that of the Sham group (Figures 2C,D).

### Impact of RDN on the circadian variations of RAS-related proteins

**Figure 3:**
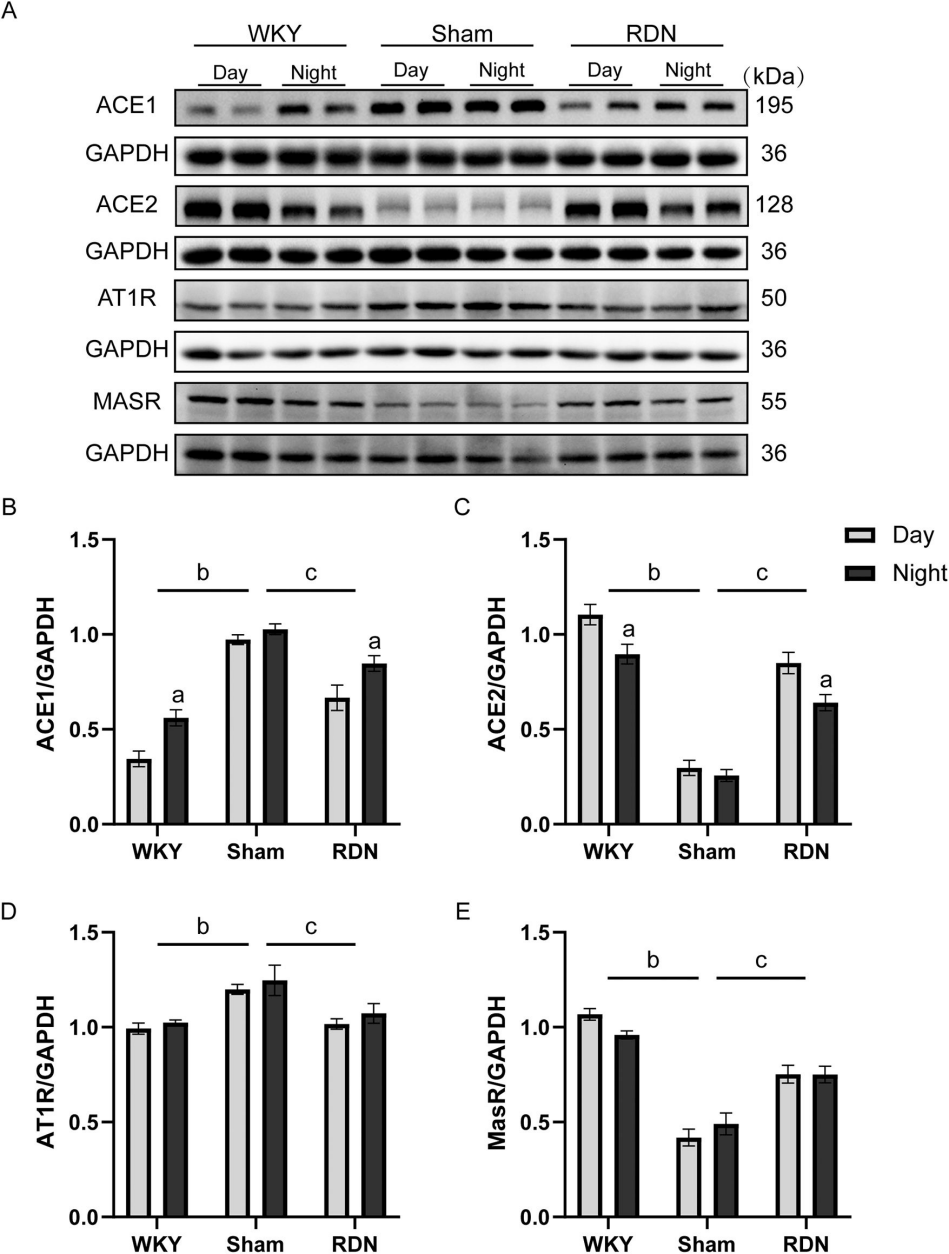
The impact of RDN on the circadian expression of renal RAS components in various groups of rats (*n*=8). **(A)** Representative images of ACE1, ACE2, AT1R, and MasR proteins, along with their quantitative analysis. **(B)** Angiotensin-converting enzyme 1 (ACE1), **(C)** Angiotensin-converting enzyme 2 (ACE2), **(D)** Angiotensin II type 1 receptor (AT1R), and **(E)** Angiotensin 1-7 receptor (MasR). Day: resting phase, Night: active phase. Compared to the resting phase (Day), ^a^ *P* < 0.05; in comparison to the overall WKY group, ^b^ *P* < 0.05; and compare to the overall Sham group, ^c^ *P* < 0.05.

In the Sham group of SHRs, renal angiotensin-converting enzyme (ACE1) levels were significantly higher than those in the WKY group, with no detectable circadian variation. In contrast, the WKY group exhibited a pronounced circadian pattern, characterized by lower levels during the day and higher levels at night. RDN significantly reduced ACE1 levels in SHRs, particularly during the resting phase (Figures 3A,B).

The Sham group also exhibited markedly lower levels of angiotensin-converting enzyme 2 (ACE2) than the WKY group, with consistently low levels during both phases. RDN significantly enhanced overall ACE2 expression, particularly restoring its levels during the resting phase (Figures 3A,C). Collectively, these findings indicate that RDN restores circadian rhythmicity of renal ACE1 and ACE2 expression in hypertensive rats.

The overall expression of angiotensin II type 1 receptor (AT1R) was significantly higher in the sham group than in the WKY group. RDN resulted in an overall reduction in AT1R expression in SHRs, with no significant circadian variation observed in any group (Figures 3A,D). Similarly, the expression of angiotensin 1–7 receptor (MasR) showed no significant circadian variation across the groups. However, the Sham group exhibited significantly lower overall MasR expression than the WKY group, whereas RDN increased MasR protein levels in SHRs (Figures 3A,E).

### Impact of RDN on the circadian variation of BMAL1 protein

**Figure 4:**
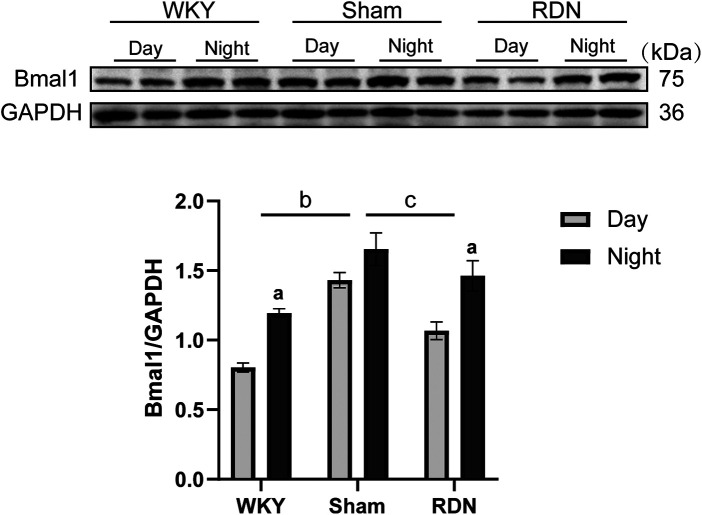
The impact of RDN on the circadian expression differences of BMAL1 (*n*=8). Representative Western blot images along with quantitative analysis are presented. Day: Resting phase; Night: Active phase. Compared to the resting phase (Day), ^a^*P* < 0.05; in comparison to the overall WKY group, ^b^*P* < 0.05; and compare to the overall Sham group, ^c^*P* < 0.05.

In WKY rats, BMAL1 expression exhibited a significant circadian rhythm, with lower levels during the day and higher levels at night. In contrast, the hypertensive sham group displayed consistently high BMAL1 expression throughout both phases with no discernible circadian variation. RDN treatment reduced daytime BMAL1 expression in hypertensive rats, thereby restoring circadian rhythmicity (Figure 4).

## Discussion

This study provides evidence that RDN not only lowers blood pressure but also ameliorates circadian BP dysregulation in spontaneously hypertensive rats. The restorative effect on BP rhythmicity was associated with concomitant stabilization of sympathetic activity rhythms, re-establishment of circadian variation in the renal renin-angiotensin system (RAS), and normalization of the oscillatory expression pattern of the core clock gene BMAL1. Our data highlight a coordinated interplay between neural, hormonal, and molecular circadian systems in hypertension and suggest RDN may confer benefits beyond absolute BP reduction.

Our finding of attenuated circadian BP variation in SHRs, which worsened with age, aligns with the clinical paradigm of non-dipper hypertension, a condition linked to elevated cardiovascular risk (20, 21). Our results are also consistent with the clinical studies that RDN effectively modulated circadian blood pressure profiles, improved circadian rhythmicity, and reduced cardiovascular risk in patients with resistant hypertension (22). This underscores that the renal nerves are not merely a conduit for tonic sympathetic drive but are integral to the temporal architecture of BP regulation. Our data imply that the therapeutic benefit of RDN may be dual-faceted: lowering mean BP and restoring its physiologic rhythm, which potentially contributing to improved long-term cardiovascular protection.

The critical role of the sympathetic nervous system in regulating the circadian rhythm of blood has been well documented (23, 24). At a mechanistic level, the stabilization of circadian sympathetic activity following RDN likely serves as a primary event. Excessive and dysregulated sympathetic output is a hallmark of hypertension and can directly disrupt peripheral circadian clocks. Our findings align with these observations by demonstrating that RDN restores circadian blood pressure rhythms in SHRs, and our study uniquely identified that this effect is associated with the modulation of sympathetic nervous system activity and its interaction with the core clock gene BMAL1. This distinction suggests a key role of sympathetic overactivation in disrupting circadian blood pressure regulation, which is a well-established contributor to hypertension. BMAL1 is essential for circadian regulation, and its role in BP control has been studied primarily in normotensive models (9, 25–27). We found that SHRs exhibit elevated renal BMAL1 expression with blunted day-night variation compared to normotensive WKY rats. RDN reduced overall BMAL1 expression and restored its circadian rhythmicity. Previous research has shown that BMAL1 knockout mice exhibit reduced expression of phenylethanolamine N-methyltransferase, a key enzyme in catecholamine synthesis, leading to decreased epinephrine and norepinephrine levels, which suggests that BMAL1 positively regulates catecholamine production, thereby linking circadian gene activity with sympathetic tone (28). In our study, changes in BMAL1 expression after RDN were associated with sympathetic stabilization, suggesting a possible bidirectional relationship. These findings provide new insight into the interaction between circadian genes and neural regulation in hypertension.

The renin-angiotensin system is a pivotal regulatory mechanism in blood pressure homeostasis and exhibits distinct circadian variations (29). Previous studies have shown that, in transgenic hypertensive (TGR) rats, plasma ACE1 activity was significantly elevated and demonstrated an inverted rhythm compared with normotensive controls (30). In rats subjected to restraint stress, Ang II infusion was shown to reverse the 24-hour blood pressure rhythm (31). Clinical studies have also highlighted the role of the RAS in circadian dysregulation; patients with non-dipper primary hypertension exhibit significantly elevated nighttime urinary angiotensinogen (U-AGT) levels, indicative of nocturnal RAS activation (32). In the present study, we observed that RDN reduced renal and plasma angiotensin II and downregulated ACE1 expression, particularly during the day, while increasing angiotensin 1–7 and upregulating ACE2 expression, especially at night. These shifts enhanced the day-night contrast in RAS components, indicating that rebalancing of the ACE1/Ang II/AT1R and ACE2/Ang1-7/MASR axes may contribute to the restoration of circadian BP variation by RDN.

It is undeniable that several methodological considerations should be acknowledged. Circadian profiling was based on two discrete time points, providing a comparative snapshot but falling short of a continuous rhythm analysis that could define precise acrophase or amplitude. Furthermore, BP was assessed via the tail-cuff method, a technique susceptible to stress-induced variability. While these limitations constrain detailed chronobiological characterization, they do not negate the consistent, directional changes observed across independent but functionally linked systems (sympathetic markers, RAS components, BMAL1). The convergence of evidence across these diverse endpoints strongly supports a genuine modulatory effect of RDN on circadian organization in hypertension. Future studies employing radiotelemetry for BP measurement and sampling across more time points are essential to fully capture the kinetics and robustness of this restoration.

The translational implications of these findings are multifaceted but require careful interpretation. Firstly, they reinforce the concept that circadian BP rhythm is a modifiable therapeutic target. Secondly, they provide a plausible multi-system mechanism for the clinical observation of improved BP rhythmicity after RDN. However, explicit extrapolation regarding the optimal timing of RDN procedures in humans based on sympathetic or molecular rhythms is not yet justified by our data. Instead, our work highlights the importance of considering circadian phenotypes in patient stratification and outcome assessment in future clinical trials. The identification of patients with significant circadian disruption may help predict those most likely to derive rhythmicity benefits from RDN. Furthermore, monitoring rhythmic biomarkers (e.g., urinary catecholamines or RAS components) could offer insights into treatment efficacy beyond 24-hour BP averages.

In conclusion, this study positions RDN as a modulator of circadian integration in hypertension. By attenuating renal sympathetic drive, RDN appears to remove a key disruptive signal that desynchronizes the local renal clock and the RAS. The subsequent restoration of BMAL1 rhythmicity and oscillatory RAS balance likely contributes to the recovery of physiological BP variation. These findings deepen our understanding of the chronopathology of hypertension and reveal the kidney as a site where neural, genetic, and hormonal circadian rhythms converge. Future research should aim to disentangle the precise sequence of these interactions and validate their relevance in human hypertension, ultimately paving the way for more personalized, rhythm-informed therapeutic strategies.

## Data Availability

The original contributions presented in the study are included in the article/Supplementary Material, further inquiries can be directed to the corresponding authors.
